# Stage Specific Assessment of *Candida albicans* Phagocytosis by Macrophages Identifies Cell Wall Composition and Morphogenesis as Key Determinants

**DOI:** 10.1371/journal.ppat.1002578

**Published:** 2012-03-15

**Authors:** Leanne E. Lewis, Judith M. Bain, Christina Lowes, Collette Gillespie, Fiona M. Rudkin, Neil A. R. Gow, Lars-Peter Erwig

**Affiliations:** 1 Division of Applied Medicine, University of Aberdeen, Aberdeen, United Kingdom; 2 Aberdeen Fungal Group, University of Aberdeen, Aberdeen, United Kingdom; University of Massachusetts Medical School, United States of America

## Abstract

*Candida albicans* is a major life-threatening human fungal pathogen. Host defence against systemic *Candida* infection relies mainly on phagocytosis of fungal cells by cells of the innate immune system. In this study, we have employed video microscopy, coupled with sophisticated image analysis tools, to assess the contribution of distinct *C. albicans* cell wall components and yeast-hypha morphogenesis to specific stages of phagocytosis by macrophages. We show that macrophage migration towards *C. albicans* was dependent on the glycosylation status of the fungal cell wall, but not cell viability or morphogenic switching from yeast to hyphal forms. This was not a consequence of differences in maximal macrophage track velocity, but stems from a greater percentage of macrophages pursuing glycosylation deficient *C. albicans* during the first hour of the phagocytosis assay. The rate of engulfment of *C. albicans* attached to the macrophage surface was significantly delayed for glycosylation and yeast-locked morphogenetic mutant strains, but enhanced for non-viable cells. Hyphal cells were engulfed at a slower rate than yeast cells, especially those with hyphae in excess of 20 µm, but there was no correlation between hyphal length and the rate of engulfment below this threshold. We show that spatial orientation of the hypha and whether hyphal *C. albicans* attached to the macrophage via the yeast or hyphal end were also important determinants of the rate of engulfment. Breaking down the overall phagocytic process into its individual components revealed novel insights into what determines the speed and effectiveness of *C. albicans* phagocytosis by macrophages.

## Author Summary

Host defence against systemic candidiasis relies mainly on the ingestion and elimination of fungal cells by cells of the innate immune system, especially neutrophils and macrophages. Here we have used live cell video microscopy coupled with sophisticated image analysis to generate a temporal and spatial analysis in unprecedented detail of the specific effects of *C. albicans* viability, cell wall composition, morphogenesis and spatial orientation on two distinct stages (macrophage migration and engulfment of bound *C. albicans*) of the phagocytosis process. The novel methods employed here to study phagocytosis of *C. albicans* could be applied to study other pathogens and uptake of dying host cells. Thus, our studies have direct implications for a much broader community and provide a blueprint for future studies with other phagocytes/microorganisms that would significantly enhance our understanding of the mechanisms that govern effective phagocytosis and ultimately the innate immune response to infection.

## Introduction

Invasive *C. albicans* infection can present a serious clinical complication, especially in patients with an impaired immune system. Host defence against systemic candidiasis relies mainly on the ingestion and elimination of fungal cells by cells of the innate immune system, especially neutrophils and macrophages [1]–[3]. Despite the clinical importance of phagocytosis, this process remains poorly understood at a mechanistic level.

The fungal cell wall is the first point of contact with the innate immune system and plays an important role in recognition and phagocytosis by host immune cells [2]. It is a dynamic, highly organized organelle that determines both the shape of the fungus and its viability. The core structure of the *C. albicans* fungal cell wall is composed of a skeleton of polysaccharide fibrils composed of β-(1,3)-glucan that is covalently linked to β-(1,6)-glucan and chitin (a β-(1,4)-linked polymer of *N*-acetylglucosamine), and is designed to function as a robust exoskeleton and a scaffold for the external glycoprotein layer [4]. This outer layer consists of highly glycosylated mannoproteins that are modified by *N*-linked and *O*-linked mannosylation and phosphomannosylation [5], [6].

Another important feature of *C. albicans* biology thought to play a major role in host recognition is the fungus' ability to undergo reversible morphological changes between yeast, pseudohyphal, and hyphal forms in response to environmental signals [7], [8]. Its morphological plasticity is considered to be the most important virulence attribute of *C. albicans* [9] and plays a major role in the fungus' capacity to successfully infect many different anatomical sites of the human host. Hyphae have invasive properties that can promote tissue penetration and escape from immune cells [10], whereas yeasts are suited to dissemination in the bloodstream [9].

Phagocytic clearance of fungal pathogens may be considered to consist of four distinct stages;(i) accumulation of phagocytes at the site where fungal cells are located; (ii) recognition of fungal pathogen-associated molecular patterns (PAMPs) through pattern recognition receptors (PRRs) (reviewed in [11]; (iii) engulfment of fungal cells bound to the phagocyte cell membrane, and (iv) processing of engulfed cells within phagocytes by fusion with lysosomal vesicles to form the phagolysosome [12].

There is very limited information on how alterations in *C. albicans* morphogenesis or cell wall composition affect phagocyte migration towards the fungus. In contrast, a significant body of literature has identified an increasing number of PRRs and downstream signalling pathways that contribute to the recognition of fungal cells by macrophages [11], [13]. These pathways have described recognition of *N*-linked mannans by the mannose receptor (MR), *O*-linked mannans by Toll-like receptor 4 (TLR4), β-glucans by dectin-1/TLR2, and α-mannosides by galectin-3/TLR2 complexes [14]. More recently, additional PRRs have been shown to contribute to *C. albicans* recognition, including the scavenger receptors CD36 and SCARF1 [15], TLR9 recognition of nucleic acids [16], dectin-2 [17] and the C-type lectin mincle [18].

Comparatively little is known about the engulfment process once the fungus is tethered to the phagocyte cell membrane. However, a series of studies have shed some light on how the overall phagocytic uptake process is affected by alterations in *C. albicans* cell wall composition, morphogenesis and macrophage activation state [10], [19]. For example, we have recently shown that the glycosylation status of the *C. albicans* cell wall profoundly affected the rate of macrophage phagocytosis. Distinct patterns emerged in that phosphomannan deficient strains (*mnn4*Δ, *pmr1*Δ, and *mnt3*Δ *mnt5*Δ) were taken up at a lower rate than the wildtype or reintegrant controls, and that *O*-linked and *N*-linked mannan deficient strains are taken up at higher rates (*mns1*Δ and *mnt1*Δ*mnt2*Δ) [10]. A study by Keppler-Ross et al. conducting competitive phagocytosis experiments suggested that macrophages displayed strong preferences for phagocytosis based on genus, species and morphology. *Candida glabrata* and *Saccharomyces cerevisiae* were taken up by J774 macrophage cells more rapidly than *C. albicans*, and *C. albicans* yeast cells were favoured over hyphal cells [20].

These studies are informative but are limited in that they assess phagocytosis in its entirety and do not break down any observed differences into individual stages of the process, such as migration, recognition or engulfment, which may be affected differentially. Furthermore, such studies assess uptake at selected time points, rather than as a continuous dynamic process, with the inherent disadvantage of ignoring temporal differences in individual stages of the phagocytosis process, which are likely to play a major role in determining the overall outcome of pathogen-host interactions *in vivo*.

Here we have conducted a comprehensive analysis of *C. albicans* phagocytosis by primary macrophages and macrophage cell lines, employing video microscopy, coupled with sophisticated image analysis tools. To assess the contribution of *C. albicans* cell wall glycosylation and the ability to switch from yeast to hyphal forms, we have taken advantage of a large collection of genetically and phenotypically characterized isogenic mutants of *C. albicans*, depleted in specific cell wall components or impaired in morphogenic switching. We show here for the first time a detailed minute by minute account of the specific effects of *C. albicans* viability, cell wall composition, morphogenesis and spatial orientation on two distinct stages (macrophage migration and engulfment of bound *C. albicans*) of the phagocytosis process. These analyses revealed that macrophage migration towards *C. albicans* was dependent on the glycosylation status of the fungal cell wall, but not cell viability or morphogenic switching from yeast to hyphal forms. Macrophages rapidly engulfed viable and UV-killed *C. albicans*, but engulfment was protracted for all glycosylation and morphogenetic mutants examined. Engulfment of hyphal *C. albicans* was determined by multiple components including hyphal length and spatial orientation.

## Results

### Macrophage migration towards *C. albicans* is affected by fungal cell wall glycosylation but not morphogenesis

*C. albicans* phagocytosis by macrophages is dependent on the *C. albicans* cell wall glycosylation status [10], but the question remains whether differences observed in overall uptake are a consequence of changes in migration of macrophages towards *C. albicans* or alterations in the engulfment process itself. Live cell video microscopy enabled examination of the individual stages of the uptake process. Representative videos are available to view in Supporting Videos S1 and S2. First we addressed the question of whether alterations in *C. albicans* cell wall glycosylation and morphogenesis affect migration of macrophages towards *C. albicans*. Primary macrophages and macrophage cell lines were challenged with glycosylation and morphogenesis defective strains of *C. albicans*. The strains used in this study are shown in Table 1. Briefly, the *mnt1*Δ*mnt2*Δstrain is deficient in *O*-glycosylation [21] and has only a single *O*-linked mannose sugar. The *mns1*Δ strain has an *N*-glycosylation defect due to curtailed α1, 2-mannosidase activity in the endoplasmic reticulum [22] and the *mnn4*Δ strain has a complete loss of phosphomannan [23]. Morphogenesis defective strains included the *hgc1*Δ strain, a G1 cyclin mutant that is unable to form true hyphae, and *efg1*Δ that lacks a specific transcription factor that regulates yeast-hypha morphogenesis pathways [24], [25].

**Table 1:** *C. albicans* strains used during this study.

Description of strain	Strain	Defect	Reference
**Reference control**			
CAI4+CIp10	NGY152		[45]
**Glycosylation mutants**			
*mnt1*Δ*mnt2*Δ	NGY111	*O*-mannosylation	[21]
*mnt1*Δ*mnt2*Δ:*MNT1*	NGY335		[21]
*mns1*Δ	HMY5	α1,2 mannosidase, terminal stage *N*-mannosylation	[22]
*mns1*Δ::*MNS1*	HMY6		[22]
*mnn4*Δ	CDH15	Phosphomannan synthesis	[23]
*mnn4*Δ:*MNN4*	CDH13		[23]
**Morphogenesis mutants**			
*hgc1*Δ	WYZ12.2	G1 cyclin/yeast-hypha morphogenes	[24]
*efg1*Δ	CA79	cAMP pathway/yeast-hypha morphogenes	[25]

**Figure 1:**
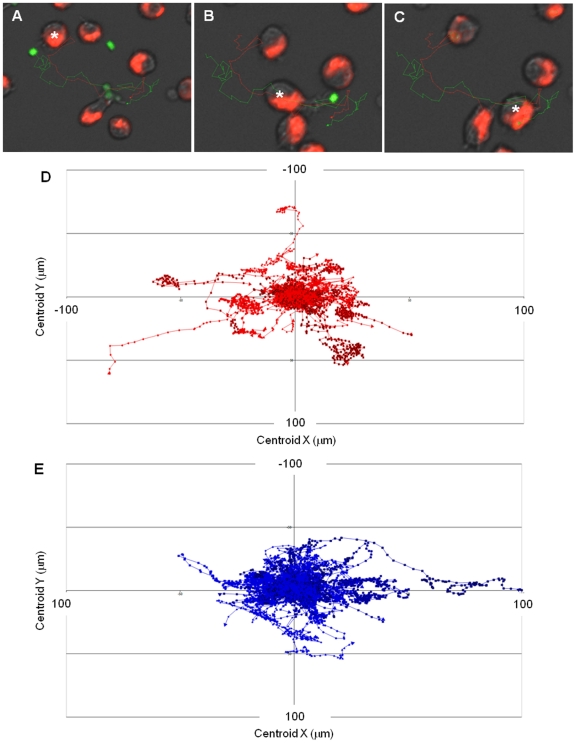
Macrophage migration towards *C. albicans*. Differences in macrophage velocity were observed when macrophages were cultured with different *C. albicans* glycosylation mutants. Tracking software was used to conduct a detailed dissection of macrophage migration dynamics. Figures 1A, B and C are snapshots from a tracking movie showing an individual macrophage (red, *) in pursuit of live wildtype *C. albicans* (green). Initially, the macrophage's movement appeared to be random (1A). After the macrophage had sensed *C. albicans*, it accelerated and homed in on its target (1B) leading to engulfment (1C). Figures 1D and E show tracking diagrams illustrating the distances travelled, directionality and velocity of J774.1 macrophages cultured with live wildtype and *mnt1*Δ*mnt2*Δ glycosylation mutant, respectively. Due to the large number of macrophages tracked per video, the data was filtered to show only active macrophages with a mean track velocity greater than 1.80 µm/min. Tracks represent the movement of individual macrophages relative to their starting position, symbols indicate the location of macrophages at 1 min intervals and arrows represent directionality.

Migration of macrophages was assessed by live cell video microscopy using our standard phagocytosis assay 10,26, with track measurements taken at 1 min intervals over a 6 h period. Figures 1A, B and C show images derived from video microscopy depicting the track of a single macrophage migrating towards and engulfing live *C. albicans* (wildtype strain). Initially, the macrophage's movement appeared to be random (Figure 1A). However, dynamic analysis suggested that macrophages sensed *C. albicans*, accelerated and homed in on their target (Figure 1B), leading to cell-cell contact and engulfment (Figure 1C). The corresponding video is available to view in Supporting Video S3. Visual inspection of the videos suggested enhanced macrophage migration towards *C. albicans* glycosylation mutants, in particular the *mnt1*Δ*mnt2*Δstrain, compared to wildtype control.

The suggestion that migration was enhanced in macrophages exposed to the *mnt1*Δ*mnt2*Δ mutant strain was further supported by macrophage tracking diagrams (Figures 1D and 1E). Tracking diagrams (Figures 1D and E) illustrate the distances travelled, directionality and velocity of macrophages cultured with live wildtype and *mnt1*Δ*mnt2*Δ, respectively. Due to the large number of macrophages tracked per video, the data were filtered to show only macrophages with a mean track velocity greater than that of inactive macrophages not pursuing fungal cells (1.80 µm/min). Tracks represent the movement of individual macrophages relative to their starting position, symbols indicate the location of macrophages at 1 min intervals and arrows represent directionality. These diagrams illustrate that although macrophages can migrate rapidly and for long distances when cultured with both live wildtype and the *mnt1*Δ*mnt2*Δ mutant, when incubated with live *mnt1*Δ*mnt2*Δ a higher number of macrophages have a mean track velocity of greater than 1.80 µm/min (Figure 1E).

**Figure 2:**
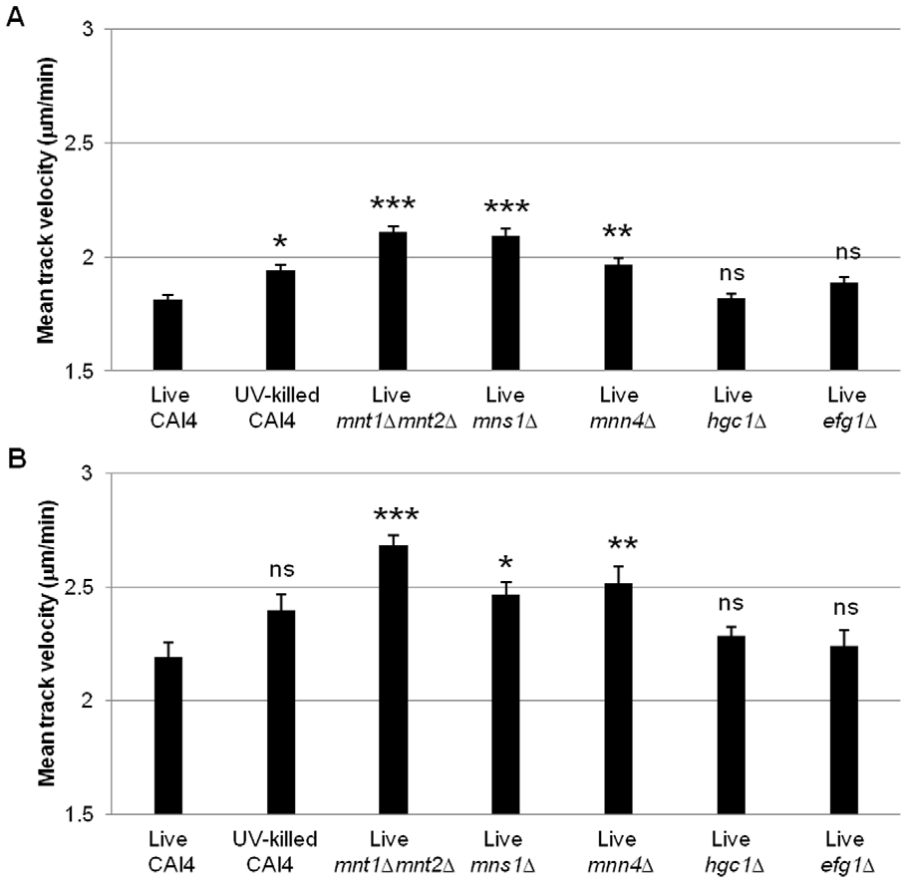
Macrophage migration towards wildtype and mutant *C. albicans*. Figures 2A and B show the mean track velocity+SE (µm/min) of J774.1 macrophages when cultured with *C. albicans* glycosylation and hyphal deficient mutants at 360 and 30 min time points, respectively. ns, not significant; *, p<0.05; **, p<0.01; ***, p<0.001.

Quantitative analysis of average macrophage track velocity for the entire length of the observation period (6 h) showed no significant differences between wildtype (1.8 µm/min ± 0.02 SE) and yeast-locked morphogenetic mutants, but confirmed enhanced migration with UV-killed wildtype *C. albicans* (1.94±0.02 SE, p<0.05) and the glycosylation mutants *mnt1*Δ*mnt2*Δ (2.1±0.02 SE, p<0.001), *mns1*Δ (2.09±0.03 SE, p<0.001) and *mnn4*Δ (1.96±0.03 SE, p<0.01) (Figure 2B). The macrophage average track velocity was highest for the first 30 min of the phagocytosis assay (Figure 2B). The data for this period again showed increased average track velocity for the glycosylation mutant strains *mnt1*Δ*mnt2*Δ (2.68±0.04, p<0.001), *mns1*Δ (2.47±0.05, p<0.05) and *mnn4*Δ (2.52±0.07, p<0.01) when compared with wildtype (2.19±0.07) (Figure 2A). In contrast, there was no significant difference in the mean track velocity of macrophages when incubated with morphogenesis defective mutants and UV-killed wildtype. Overall track lengths were measured for the first 30 min and 6 h, and not surprisingly the data reflected the average track velocity for the wildtype and mutant strains tested (data not shown). Enhanced macrophage migration in phagocytosis assays with *C. albicans* glycosylation mutants was not a consequence of alterations in maximal macrophage velocity (average max velocity in µm/min: wildtype, 3.7±0.2; *mnt1*Δ*mnt2*Δ, 3.6±0.1; *mnn4*Δ, 3.6±0.2; *mns1*Δ (3.1±0.2) but rather a reflection of increased macrophage activity, particularly during the first hour of the interaction assay.

Experiments with primary murine peritoneal macrophages showed a similar pattern. We observed no differences between the yeast-locked mutant strain *hgc1Δ* and wildtype but significantly increased average track velocity for the glycosylation mutant strain *mnt1*Δ*mnt2*Δ (p<0.001) for the first 30 min and 6 h of the phagocytosis assay (Table 2). Overall the mean track velocity of peritoneal macrophages was found to be significantly lower than for J774.1 macrophages (p<0.001). However, the maximum velocity of peritoneal macrophages (3.9±0.2 µm/min) was comparable to J774.1 macrophages (3.7±0.2 µm/min). We hypothesised that the difference in mean track velocity between peritoneal and J774.1 macrophages is due in part to the peritoneal macrophages being more spread out and covering a larger surface area, therefore, reducing the need to migrate to achieve close proximity with fungal cells in the phagocytosis assay. However, experiments using the same macrophage:*C. albicans* ratios but lower macrophage densities confirmed that this was not the case, as mean track velocities were unchanged (Table 2). Thus, changes in *C. albicans* cell wall composition but not hyphal morphogenesis markedly influenced macrophage migration *in vitro*.

**Table 2:** Mean track velocity of peritoneal macrophages exposed to *C. Albicans*.

*C. albicans* strain	Mean track velocity (mm/min) at 30 min	Mean track velocity (mm/min) at 360 min
CAI4+CIp10	1.88±0.012	1.39±0.009
Sparce CAI4+CIp10	1.82±0.014	1.24±0.009
*hgc1*Δ	2.02±0.012	1.59±0.010
*mnt1*Δ*mnt2*Δ	2.53±0.013*	1.97±0.011*
*mnt1*Δ*mnt2*Δ::*MNT1*	2.01±0.013	1.44±0.009

Table 2 shows the mean track velocity+SE (µm/min) of peritoneal macrophages cultured with *C. albicans* glycosylation and hyphal deficient mutants at 30 and 360 min time points, respectively.

*: ,p<0.001.

### Macrophages rapidly engulfed viable and UV-killed *C. albicans* but engulfment was delayed for glycosylation and yeast-locked mutants

**Figure 3:**
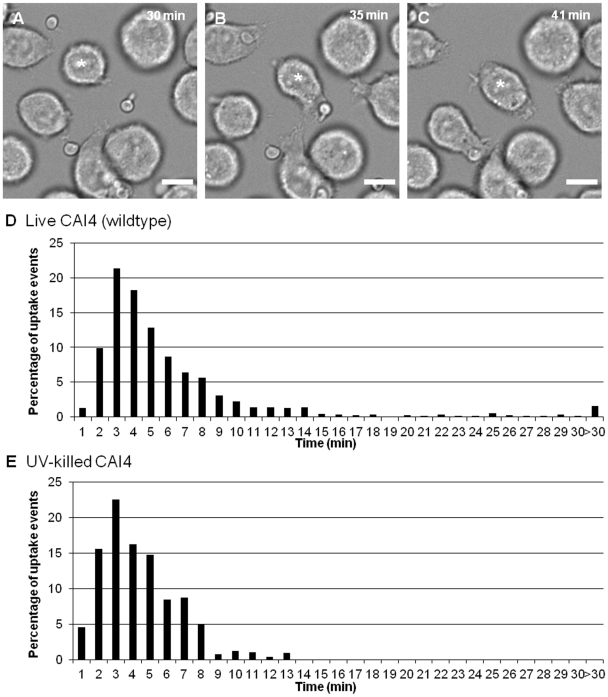
Macrophage engulfment of live and UV-killed wildtype *C. albicans*. Figures 3A, B, and C are snapshots taken from live cell video microscopy capturing the engulfment process. Figure 3A shows a macrophage (*) and *C. albicans* prior to cell-cell contact, Figure 3B shows the same cells during contact and Figure 3C shows *C. albicans* within the macrophage post engulfment. Scale bar, 10 µm. Figures 3D and 3E show the time taken for J774.1 macrophages to ingest live and UV-killed wildtype *C. albicans* following cell-cell contact verses the percentage of uptake events, respectively (n = 6). The majority of live *C. albicans* were engulfed rapidly by macrophages once cell-cell contact was established (Figure 3D). However, UV-killed *C. albicans* were engulfed more swiftly, with all cells internalised within 15 min (Figure 3E).

Effective migration of macrophages towards *C. albicans* is necessary to establish cell-cell contact, which is a prerequisite for initiation of the engulfment process. Next we addressed the question of whether alterations in *C. albicans* cell wall glycosylation and morphogenesis affected the ability and speed by which macrophages engulfed *C. albicans* after cell-cell contact was established. Live cell video microscopy coupled with image analysis generated a detailed minute by minute account of the engulfment process (Figures 3A, B and C). Wildtype *C. albicans* was shown to be rapidly engulfed by macrophages once cell-cell contact was established (Figure 3D). A three dimensional projection image confirming *C. albicans* phagocytosis is available in Supporting VideoS4.The average time taken for engulfment of wildtype *C. albicans* is 6.7±0.3 min, and the vast majority (95%) of fungal cells were engulfed within 15 min. UV-killed *C. albicans* yeast cells were engulfed even more swiftly, with all cells taken up within 15 min and engulfment taking an average of 4.2±0.1 min (Figure 3E).

**Figure 4:**
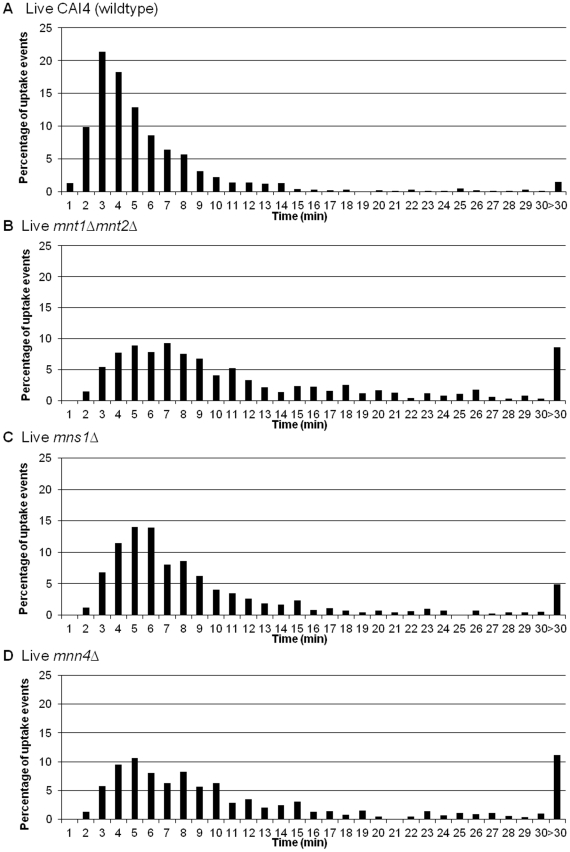
Macrophage engulfment of wildtype and glycosylation mutants of *C. albicans*. Figures 4A, B, C and D show the time taken for J774.1 macrophages to ingest live wildtype (n = 6), *mnt1*Δ*mnt2*Δ (n = 6), *mns1*Δ (n = 3) and *mnn4*Δ (n = 3) strains following initial cell-cell contact verses the percentage of uptake events. The rate of engulfment of all glycosylation mutant strains (Figures 4B–D) was significantly slower than that of wildtype *C. albicans* (Figure 4A).

**Figure 5:**
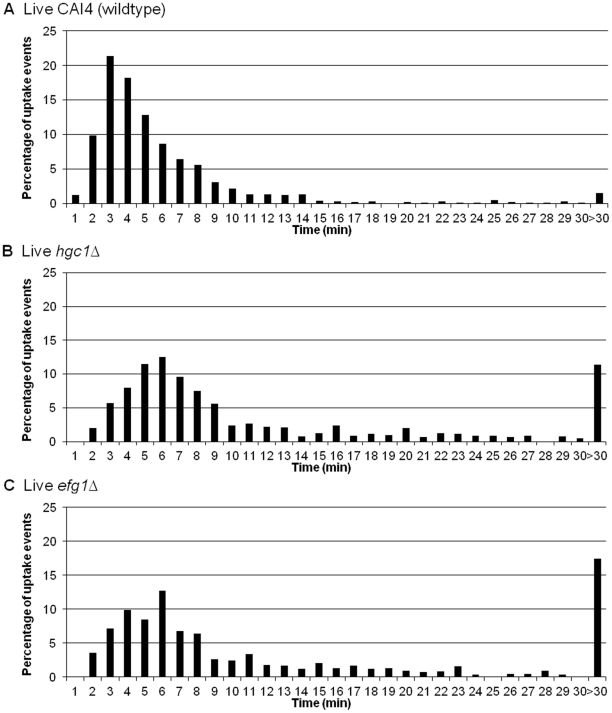
Macrophage engulfment of wildtype and yeast-locked morphogenetic mutant strains of *C. albicans*. Figures 5A, B and C show the time taken for J774.1 macrophages to ingest live wildtype (n = 6), *hgc1*Δ(n = 6) and *efg1*Δ (n = 3) following initial cell-cell contact plotted versus the percentage of uptake events. The average time taken for engulfment of the *hgc1*Δ (Figure 5B) and *efg1*Δ (Figure 5C) mutant strains was significantly greater than for the wildtype control (Figure 5A).

Interestingly, the rate of engulfment of all glycosylation mutant strains (Figures 4B–D) was significantly slower than that of wildtype *C. albicans* (*mnt1*Δ*mnt2*Δ) (p<0.001); *mns1*Δ (p<0.01; *mnn4*Δ (p<0.001) (Figure 4A). The delayed engulfment was most marked for the *mnt1*Δ*mnt2*Δ (13.5±0.7 min) and *mnn4*Δ (14.4±0.9 min) mutant strains (Figures 4B and D), as macrophages on average took twice as long to engulf these mutants than wildtype *C. albicans* (Figure 4A). Control strains *mnt1*Δ*mnt2*Δ::*MNT1* and *mnn4*Δ::*MNN4*, containing a single reintegrated copy of the corresponding deleted genes, partially restored the ability of macrophages to swiftly engulf *C. albicans* (data not shown). Experiments using primary thioglycollate elicited murine peritoneal macrophages and human monocyte derived macrophages also showed a significant delay for the engulfment of the glycosylation deficient mutant *mnt1*Δ*mnt2*Δ and this was partially restored in the corresponding reintegrant control *mnt1*Δ*mnt2*Δ::*MNT1(* *Table 3* *)*. Engulfment of yeast-locked morphogenetic mutant strains was delayed and ultimately impaired, relative to wildtype controls in J774 macrophages (p<0.001). Firstly, the average time taken for engulfment of the *hgc1*Δ (Figure 5A and B) and *efg1*Δ (Figure 5A and C) mutant strains was significantly greater than for the wildtype control (16.2±1.4 min, 21.9±2.4 min and 6.7±0.3 min, respectively). Engulfment of approximately 1.5% of wildtype *C. albicans* took longer than 30 min, compared with approximately 11% and 17% for *hgc1*Δ and *efg1*Δ, respectively.

**Table 3:** Engulfment of wildtype and mutant of *C. albicans by p*rimary macrophages.

*C. albicans* strain	Average time for engulfment (min) by peritoneal macrophages	Average time for engulfment (min) by human monocyte derived macrophages
CAI4+CIp10	8.56±2.56	6.9±0.32
UV killed CAI4+CIp10	7.39±0.24	-
*hgc1*Δ	9.85±3.14	-
*mnt1*Δ*mnt2*Δ	11.9±1.1*	8.5±0.28*
*mnt1*Δ*mnt2*Δ::*MNT1*	7.41±0.67	-

Table 3 shows the average time taken+SD (min) for peritoneal macrophages and human monocyte derived macrophages to engulf wildtype and mutant *C. albicans*. N = 3,

*: ,p<0.01.

Secondly, we observed that approximately 2% of *hgc1*Δ and 41% of *efg1*Δ mutants that established contact with macrophages were not internalised, even after prolonged cell-cell contact. In contrast, all wildtype *C. albicans* yeasts were successfully engulfed following recognition. However, 66% of the *efg1*Δ mutant cells were eventually engulfed by neighbouring phagocytes after detachment from the macrophage they were originally in contact with. Experiments using peritoneal macrophages showed a non significant delay in the engulfment of the yeast locked mutant strain hgc1Δ (Table 3) and little evidence of detachment once cell-cell contact was established.

Thus, macrophages rapidly engulfed viable and UV-killed *C. albicans* after cell-cell contact was established, but engulfment was markedly slower for all glycosylation (in all macrophage subsets studied) and yeast-locked (solely in the J774 macrophage cell line) morphogenetic mutants examined.

### Macrophages are more effective at engulfing yeast rather than hyphal *C. albicans* and engulfment is influenced by hyphal length

**Figure 6:**
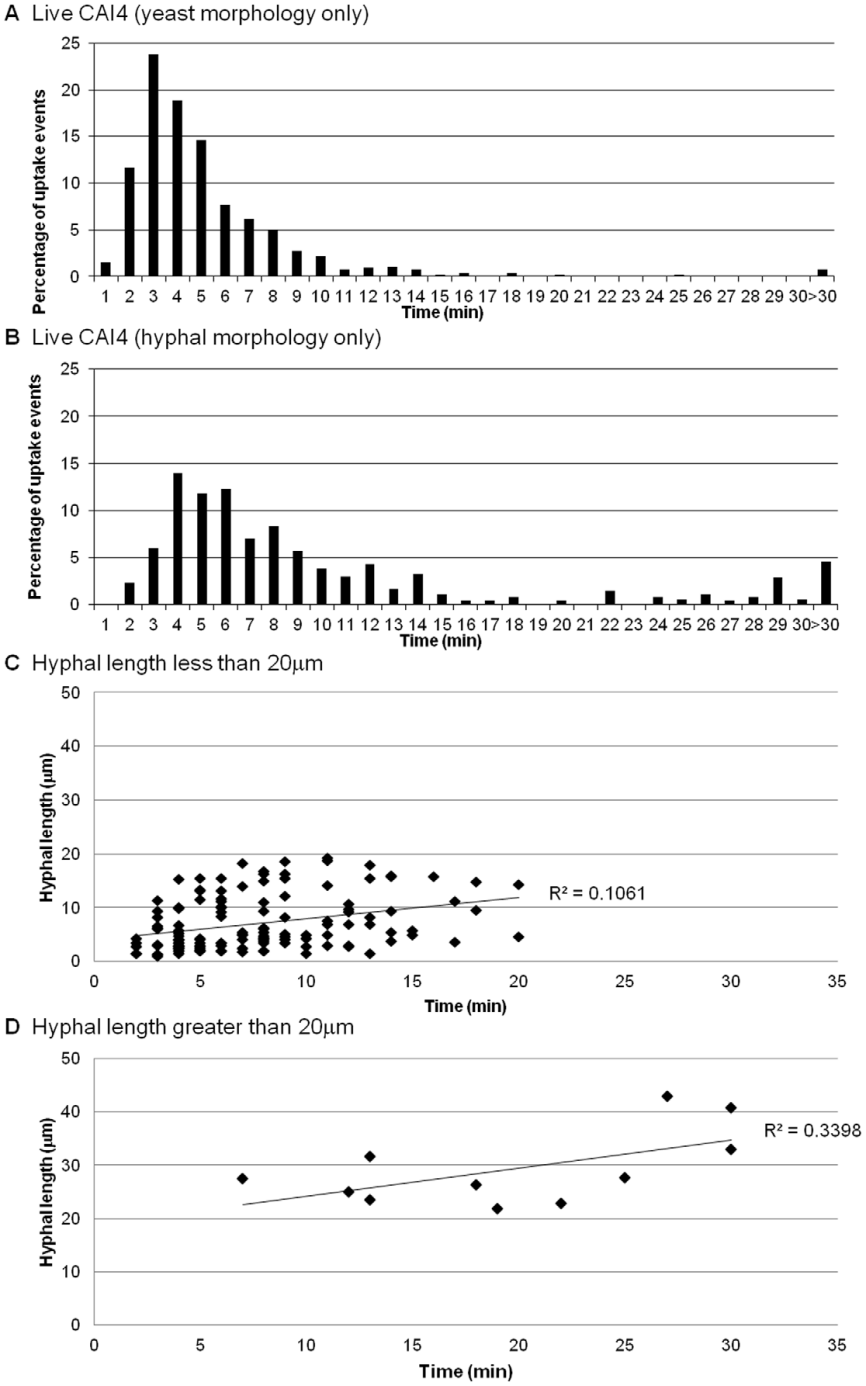
Role of hyphal morphogenesis in the engulfment of *C. albicans* by macrophages. Figures 6A and B show the time taken for J774.1 macrophages to ingest live *C. albicans* in yeast and hyphal morphology plotted against the percentage of uptake events (n = 6). Hyphal *C. albicans* was engulfed at a significantly slower rate than the yeast form of *C. albicans*. Figures 6C and D show the time taken for macrophages to engulf *C. albicans* with hyphae less than and greater than 20 µm, respectively (n = 6). There was no correlation between hyphal length and speed of engulfment for hyphal *C. albicans* of less than 20 µm length (Figure 6C). However, when hyphal length exceeded 20 µm there was significant impact on the macrophage's ability to engulf *C. albicans* (Figure 6D).

The data above showed that UV-killed yeast cells were engulfed more rapidly than live wildtype cells that were able to form hyphae. The accelerated engulfment of UV-killed cells raised questions about how cell morphology affects engulfment of *C. albicans* by macrophages. We examined the engulfment of wildtype *C. albicans* cells that had established cell-cell contact with macrophages in either yeast or hyphal morphology. Hyphal *C. albicans* cells were engulfed at a slower rate than *C. albicans* yeast cells (10.8±0.9 min and 5.6±0.3 min, respectively). Furthermore, the vast majority (98%) of yeast cells of *C. albicans* were taken up within 15 min (Figure 6A), whereas there was greater variability for hyphal cells of *C. albicans*, with 21% *taking* longer than 15 min to become engulfed (Figure 6B).

Next we examined whether hyphal length influenced the speed of engulfment, perhaps explaining the variations observed for hyphal *C. albicans* engulfment. Macrophages were capable of ingesting *C. albicans* with hyphae of more than twice the average diameter of macrophages (the maximum observed length of ingested hyphae was 42.9 µm), but the mean hyphal length at time of engulfment was 8.7±0.7 µm. Intriguingly, and contrary to expectations, we found no correlation between hyphal length and speed of engulfment for hyphal cells of *C. albicans* of less than 20 µm length (Figure 6C). However, when hyphal length exceeded 20 µm there was as significant impact on the macrophage's ability to engulf *C. albicans* (Figure 6D). Although macrophages engulfed *C. albicans* with hyphae larger than 20 µm, uptake was markedly slower with 64% of uptake events requiring more than 15 min. It is worth noting that despite having difficulty engulfing large hyphae macrophages were nonetheless persistent in their attempt to do so. Thus, macrophages were more effective at engulfing yeast cells rather than hyphal cells of *C. albicans* and engulfment of hyphal cells was influenced in part by hyphal length, with a cut off of 20 µm, above which macrophage engulfment was markedly impaired.

### The rate of engulfment of hyphal cells of *C. albicans* was influenced by spatial orientation

**Figure 7:**
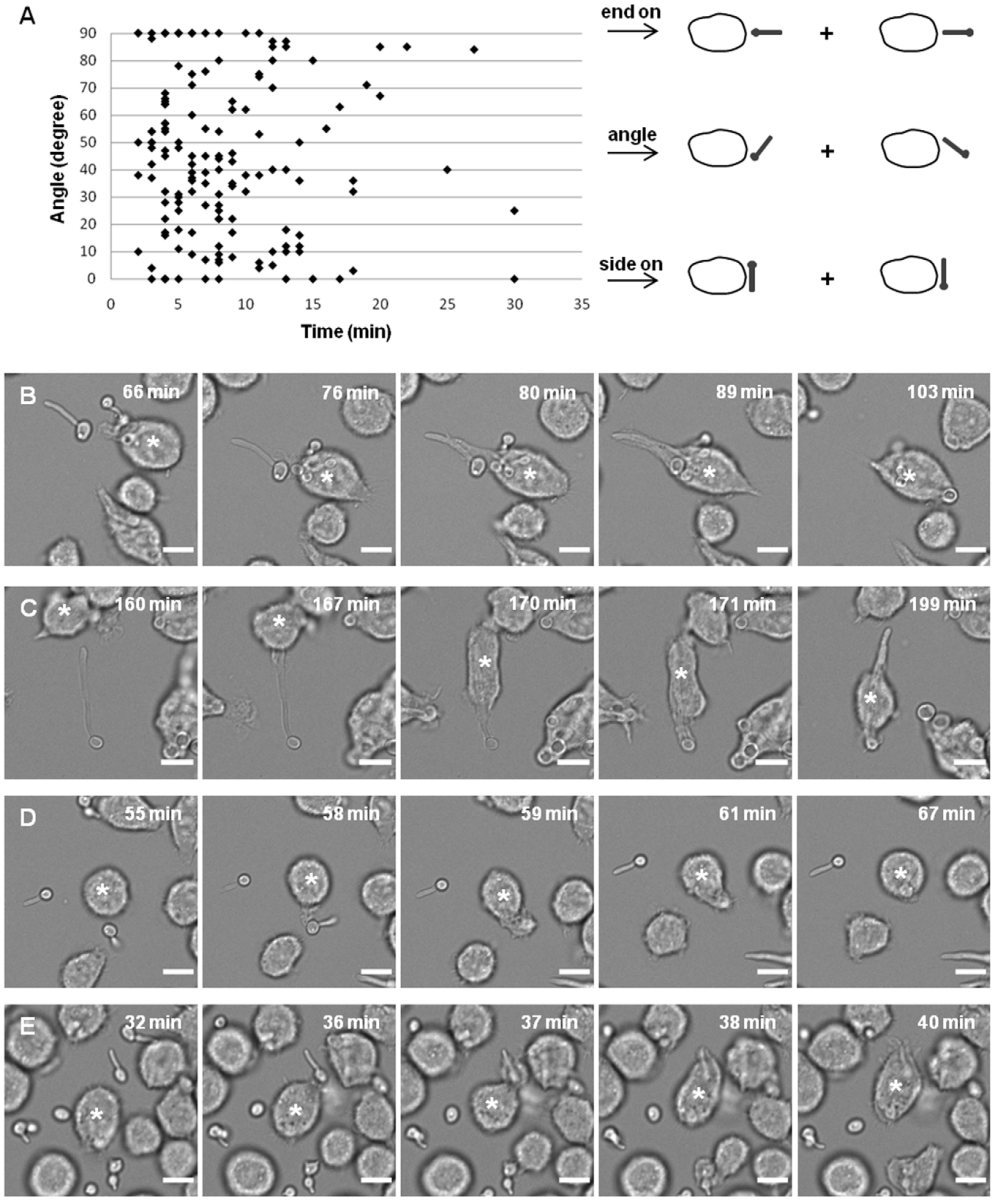
The role of *C. albicans* spatial orientation in engulfment by J774.1 macrophages. Figure 7A plots the time taken for engulfment of individual hyphal wildtype *C. albicans* in relation to the exact angles at which cell-cell contact was established (0°, side-on; 90°, end-on). *C. albicans* can be engulfed in any spatial orientation, including end-on, side-on and at an angle (Figure 7A). Figures 7B–E show snapshots from live cell video microscopy movies showing *C. albicans* being taken up in a variety of orientations, including yeast end on, (Figure 7B), germ tube apex end-on (Figure 7C), side-on (7D) and at an angle (7E). Scale bar, 10 µm; macrophage of interest, *.

Finally, we took advantage of the large quantity of data amassed from live cell video microscopy phagocytosis assays to address previously unanswered questions relating to how spatial orientation of *C. albicans* may affect the efficiency of engulfment by macrophages. First, we established that hyphal cells of *C. albicans* could be taken up by macrophages independent of their spatial orientation (Figure 7A). *C. albicans* germ tubes could be engulfed yeast-end on and germ tube apex-end on (Figures 7B and C), side-on (Figure 7D) and at an angle (Figure 7E). However, it is noteworthy that although cell-cell contact could be initiated in any orientation, the rate of engulfment was affected; *C. albicans* that made contact in an end-on orientation were taken up more rapidly than those engulfed at an angle or where cell-cell contact was initiated side-on (5.5±0.7 min, 8.8±0.5 min and 9.5±2.3 min, respectively). The large SE observed when *C. albicans* makes contact side-on can be explained by the fact that macrophages had particular difficulty ingesting large hyphae (>20 µm) in the side-on orientation. The end initially encountered by the macrophage appeared to be random. Approximately equal numbers of encounters occurred that were yeast end-on or hyphal end-on, but there was a propensity for C. albicans to be taken up more rapidly yeast-end on (8.0±0.6 min) than hyphal end-on (9.8±1.0 min).

Initial *C. albicans* orientation when establishing cell-cell contact with macrophages influenced the rate of engulfment with end-on contact of the hyphal end resulting in the most rapid engulfment. Thus, engulfment of hyphal cells of *C. albicans* was influenced by multiple factors including hyphal length and spatial orientation, and whether the initial encounter was by the yeast or hyphal end.

## Discussion

*C. albicans* is a major life-threatening human fungal pathogen. Host defence against systemic *Candida* infection relies mainly on phagocytosis of fungal cells by cells of the innate immune system. In this study, we analysed the contribution of distinct *C. albicans* cell wall components and yeast-hypha morphogenesis to specific stages of phagocytosis by macrophages.

We show that macrophage migration towards *C. albicans* was dependent on the glycosylation status of the fungal cell wall, but not cell viability or morphogenic switching from yeast to hyphal forms. This finding was not a consequence of differences in maximal macrophage track velocity, but stems from a greater percentage of macrophages pursuing glycosylation deficient *C. albicans* cells during the first hour of the phagocytosis assay. The rate of engulfment of *C. albicans* by macrophages was significantly slower for glycosylation and morphogenesis deficient mutant strains, but enhanced for non-viable cells. Hyphal cells were engulfed at a slower rate than yeast cells, especially those with hyphae in excess of 20 µm, but there was no correlation between hyphal length and the rate of engulfment below this threshold. We show that spatial orientation of the hypha and whether hyphal *C. albicans* attached to the macrophage via the yeast or hyphal end were also important determinants of the rate of engulfment.

This is the first study, to our knowledge, to show that individual stages of *C. albicans* phagocytosis by macrophages are differentially affected by changes in *C. albicans* cell wall composition. Our previous work, using assays that globally assess phagocytosis, have shown increased phagocytosis of *O*-linked and *N*-linked mannan deficient strains (*mns1*Δ and *mnt1*Δ *mnt2*Δ) [10]. Intriguingly, we show here that changes in cell wall glycosylation enhance macrophage migration towards *C. albicans*, but delay engulfment once cell-cell contact is established. This illustrates that standard assays do not differentiate between the individual stages of the phagocytosis process and are unable to detect significant temporal differences in migration or engulfment. For example, we show here that phosphomannan deficient cells of *C. albicans* were engulfed less efficiently. This effect was much less obvious in previous studies that simply evaluated phagocytosis efficiency by single time point measurements [27]. Macrophage migration was enhanced in all glycosylation mutants but most markedly in the *mnt1*Δ*mnt2*Δ *O*-glycosylation mutant. This translated into much higher overall uptake compared to the phosphomannan deficient *mmn*4Δ mutant and is in keeping with our previous published results [10]. It is conceivable that enhanced macrophage migration in response to the absence of *O*-linked (*mnt1*Δ*mnt2*Δ) or *N*-linked mannans (*mns1*Δ) is a consequence of unmasking underlying β-glucans [28], [29] or electrostatic signals as a consequence of alterations in surface charge following loss of phosphomannan (*mnn4*Δ) [30], [31]. Observation of individual macrophage migration patterns indicated that macrophage movement was slow and random initially, but became directional towards a specific *C. albicans* cell, associated with a marked increase in macrophage velocity. Macrophage acceleration towards *C. albicans* occurred at distances in excess of 15 µm and, therefore, suggests the presence of a chemotactic signal. Key candidates are a number of glycolipids that are known to be shed by *C. albicans* and are potent inducers of macrophage cytokine synthesis *in vitro* and *in vivo* [32]. We are currently conducting detailed mathematical modelling of the macrophage tracking patterns to further elucidate the hypothesis that macrophage migration towards *C. albicans* is affected by differences in shedding of glycolipids between wildtype and glycosylation deficient strains.

We have shown that macrophage uptake of *C. albicans* is a multi-step process, involving recognition and subsequent engulfment of *C. albicans*. *C. albicans* cell wall mannosylation is a key determinant in the rate of engulfment; the absence of specific PAMPs in the glycosylation mutant strains delays engulfment once cell-cell contact has been established, and this may be a consequence of differential activation of macrophage PRRs. This is in keeping with experiments in which *C. albicans* cell wall mutants were combined with specific macrophage receptor blocking methods that have been used to define the PAMP-PRR interactions required for cytokine induction [14], [33]. These *in vitro* findings are relevant to *C. albicans* infections *in vivo*, since *C. albicans* mutants with defects in cell wall mannosyl residues are also less virulent in experimental models of disseminated candidiasis [21], [22], [34]–[36].

Morphological plasticity is one of the hallmarks of the human fungal pathogen *C. albicans* [37], and its ability to switch between yeast and hyphal forms is thought to contribute to pathogenesis [24], [25], [38], [39]. *C. albicans* mutants that are unable to form filaments are less virulent [40], although conversely, mutants that are unable to grow as yeast are also less virulent [41]. There are conflicting reports in the literature regarding the efficiency of macrophage phagocytosis for *C. albicans* yeast and hyphal forms [20], [42], [43]. Here we show definitively data supporting the notion that macrophages are more effective at engulfing *C. albicans* yeasts. Furthermore, the use of video microscopy coupled with thorough analysis of large numbers of individual macrophage-*C. albicans* interactions provides a minute-by-minute account of the engulfment process, which offers detail that has not been previously available. A prime example is our observation that yeast-locked *C. albicans* cells were engulfed less efficiently than wildtype *C. albicans*. Not only was this not obvious in previous studies that simply evaluated phagocytosis efficiency by single time point measurements [10], but in addition, we observed here that delayed engulfment can result in detachment of the fungal cell and engulfment by a neighbouring macrophage. One may speculate that *in vivo* where phagocyte numbers are limited this may have a significant impact on pathogen clearance and that yeast locked mutant *C. albicans* cells have properties other than the induced phenotype that differ from wildtype yeast *C. albicans* cells. However, the observed delay in engulfment for yeast-locked mutant *C. albicans* in the macrophage cell line was almost completely abrogated in experiments using primary macrophages, underlining the importance of studying host-pathogen interactions in multiple phagocyte subsets.

The approach taken here further enabled us to dissect the complexity of engulfment of hyphal *C. albicans* by macrophages. We show that macrophages are capable of engulfing hyphal *C. albicans* in excess of 40 µm (approximately twice the diameter of macrophages) - in keeping with reports that macrophages are capable of ingesting apoptotic epithelial cells in the involuting mammary gland of similar or even larger size [44]. Hyphal length, however, does play a major role in the engulfment process of *C. albicans*, in that engulfment of *C. albicans* with hyphae in excess of 20 µm took significantly more time and phagocytosis was frequently frustrated. Interestingly, we show that below a 20 µm hyphal length threshold there was no correlation between hyphal length and the rate of engulfment. These observations are most likely related to difficulties associated with macrophages attempting to engulf very large particles. In addition to hyphal length, we have identified two other factors that influenced engulfment of hyphal *C. albicans*. We showed that the rate of engulfment was determined by the orientation in which *C. albicans* was encountered, with end-on being favourable to side-on orientation, suggesting that steric hindrance affects engulfment. We also showed that yeast end-on engulfment was more efficient than hyphal end-on encounters. This in turn may reflect differences in the wall chemistry of the hyphal tip compared to the mother cell, or be due to the efficiency of the assembly of proteins of the phagocytic cup for objects of different sizes and shapes [45].

Here we have conducted the most detailed analysis of the contribution of *C. albicans* viability, cell wall glycosylation and morphogenesis to phagocytosis by macrophages to date, to our knowledge. Our approach of combining live cell video microscopy with image analysis tools for the migration analysis, and minute-by-minute analysis of thousands of individual macrophage-*C. albicans* interactions, provides unique insight into the complexity of *C. albicans* phagocytosis by macrophages. The novel methods employed here to study phagocytosis of *C. albicans* could be applied to study other pathogens and uptake of dying host cells. Such studies would significantly enhance our understanding of the mechanisms that govern effective phagocytosis and ultimately the innate immune response to infection.

## Materials and Methods

### Ethics statement

All animal experiments have been conducted in strict accordance with UK Home Office guidelines. The appropriate project and personal licenses are in place PIL 60/6194 and approved by the UK Home office.

### *C. albicans* strains and growth conditions

*C. albicans* serotype A strain CAI4+CIp10, hitherto referred to as the parental wildtype, was used as a control and its parent strain, CAI4, was used to construct mutants using targeted gene disruption [46]. The mutants used are listed in Table 1. *C. albicans* strains containing a single reintegrated copy of the corresponding deleted genes to regenerate the heterozygous genotype acted as controls. Most of the *C. albicans* strains used were created in house and have been described previously [21]–[25]. *C. albicans* strains were obtained from glycerol stocks stored at −80°C, and plated on SC-Ura plates (except *hgc1*Δand *efg1*Δ). SC-Ura plates consist of 6.9 g yeast nitrogen base without amino acids (Formedium, Norfolk, UK), 1 ml 1 M NaOH (BDH Chemicals, VWR International, Leicestershire, UK), 10 ml 1% (w/v) adenine hemisulphate salt (Sigma, Dorset, UK), 50 ml 40% D-glucose (Fisher Scientific, Leistershire, UK), 50 ml 4% SC-Ura dropout (Formedium, Norfolk, UK) and 2% (w/v) technical agar (Oxoid, Cambridge, UK) made up to 1000 ml in distilled H2O. The *C. albicans* morphogenetic mutants *hgc1Δ* and *efg1Δ* were grown on YPD plates consisting of 1% yeast extract (Duchefa Biochemie, Haarlem, Holland), 2% mycopeptone (Oxoid, Cambridge, UK), 2% D-glucose and 2% technical agar in distilled H2O. All plates were incubated at 30°C until colonies formed, and were then stored at 5°C.

### Preparation of thioglycollate-induced peritoneal mouse macrophages

Intraperitoneal injections of 1 ml Brewer's thioglycollate broth (BD, New Jersey, USA) were administered to 8 week old female BALB/c mice. After 4 days, the peritoneal cavity of sacrificed mice was lavaged with 5 mM EDTA in 1× PBS, to harvest thioglycollate-induced macrophages. These Thio-macrophages were washed 3 times with RPMI medium 1640 (Sigma, Dorset, UK) supplemented with 10% (v/v) foetal calf serum (FCS) (Biosera, Ringmer, UK), 200 U/ml penicillin/streptomycin antibiotics (Invitrogen Ltd, Paisley, UK), 10 mM HEPES (Invitrogen Ltd, Paisley, UK) and 2 mM L-glutamine (Invitrogen, Paisley, UK). For phagocytosis assays, 1×10^6^ thio-macrophages in 2 ml supplemented RPMI medium were seeded onto glass bottomed Iwaki dishes (VWR, Leistershire, UK) and cultured overnight at 37°C with 5% CO_2_. Immediately prior to experiments, RPMI medium was replaced with 2 ml pre-warmed supplemented CO_2_-independent medium (Gibco, Invitrogen, Paisley, UK) containing 1 µM LysoTracker Red DND-99 (Invitrogen, Paisley, UK). LysoTracker Red DND-99 is a red fluorescent dye that stains macrophage acidic organelles, enabling macrophage paths to be tracked using Volocity 5.0 software (Improvision, PerkinElmer, Coventry, UK).

### Preparation of J774.1 mouse macrophage cell line and HMDM

J774.1 macrophages (ECACC, HPA, Salisbury, UK) were maintained in tissue culture flasks in DMEM medium (Lonza, Slough, UK), supplemented with 10% (v/v) FCS (Biosera, Ringmer, UK), 200 U/ml penicillin/streptomycin antibiotics (Invitrogen, Paisley, UK) and 2 mM L-glutamine (Invitrogen, Paisley, UK) at 37°C with 5% CO_2_. Human monocyte derived macrophages were prepared as previously described in detail [26]. For phagocytosis assays, 1×10^6^ J774.1 macrophages in 2 ml supplemented DMEM medium were seeded onto glass based Iwaki dishes (VWR, Leistershire, UK) and cultured overnight at 37°C with 5% CO_2_. Immediately prior to experiments, DMEM medium was replaced with 2 ml pre-warmed supplemented CO2-independent medium (Gibco, Invitrogen, Paisley, UK) containing 1 µM LysoTracker Red DND-99 (Invitrogen, Paisley, UK).

### *C. albicans* preparation and staining with fluorescein isothiocyanate (FITC)

Single *C. albicans* colonies from plates stored at 5°C were cultured in 5 ml SC-Ura/YPD medium (recipes as above, excluding technical agar) and incubated overnight at 30°C, 200 rpm. In order to determine the impact of *C. albicans* viability on macrophage migration and engulfment, wildtype *C. albicans* were killed by UV-irradiation; 100×10^6^ fungal cells in 1 ml 1× PBS were exposed to 20 doses of UV irradiation at 100 mJ/cm^2^ in 6 well plates. To aid visualisation of *C. albicans* during phagocytosis assays, 100×10^6^ live or UV-killed *C. albicans* were stained using 1 mg/ml FITC (Sigma, Dorset, UK) in 0.05 M carbonate-bicarbonate buffer (pH 9.6) (BDH Chemicals, VWR International, Leicestershire, UK) for 10 min at room temperature in the dark. Fungal cells were washed 3 times in PBS to remove unbound FITC and resuspended in 1× PBS.

### Live cell video microscopy phagocytosis assays

Our standard phagocytosis assays were performed as previously described [10]. In brief, 3×10^6^ FITC-stained *C. albicans* were added to 1×10^6^ macrophages in glass based Iwaki dishes (VWR, Leistershire, UK) immediately prior to imaging. Video microscopy experiments were performed using a DeltaVision Core microscope (Applied Precision, Washington, USA) with an environmental control chamber set at 37°C. Images were captured at 1 min intervals for 6 h using an EMCCD camera. At least two independent experiments were conducted for each *C. albicans* strain, and at least 3 movies were analysed from each experiment. One hundred macrophages were randomly selected from each movie and their phagocytic activity determined (as below).

### Analysis of live cell video microscopy movies

Volocity 5.0 imaging analysis software was used to track macrophage migration at 1 min intervals throughout the 6 h phagocytosis assay. The software enabled high throughput analysis of macrophage migration, providing detailed information on the distances travelled, directionality and velocity of thousands of individual macrophages. Data were subsequently displayed in tracking diagrams and used to calculate the mean track velocity and track length of macrophages cultured with *C. albicans*. These analyses enabled assessment of the affects of *C. albicans* viability, glycosylation status and morphology on migration.

One hundred macrophages from each movie were analysed individually at 1 min intervals throughout the 6 h phagocytosis assay. Measurements taken include the time points at which initial cell-cell contact occurred and at which *C. albicans* was fully enclosed, the number of *C. albicans* taken up and their morphology, the orientation of hyphal *C. albicans* relative to the macrophage and hyphal length. The rate of engulfment of live and UV-killed wildtype *C. albicans*, and glycosylation and yeast-locked morphogenetic mutant *C. albicans* was calculated by subtracting the time point at which initial cell-cell contact occurred from the time point at which the fungus was fully phagocytosed. This enabled accurate assessment of the affects of *C. albicans* viability, glycosylation status and morphology on the speed of engulfment. *C. albicans* spatial orientation, morphology, hyphal length and the end of hyphal *C. albicans* recognised were determined to assess whether these factors impact on the rate of engulfment. This strategy enabled in depth analysis of individual *C. albicans*-macrophage interactions in real time.

### Statistical analysis

Mean values and standard errors were calculated. One-way analysis of variance (ANOVA) and Tukey-Kramer Multiple Analysis Comparison Tests were used to determine statistical significance.

## Supporting Information

Video S1 **Phagocytosis of live C. albicans by J774.1 macrophages.** Shows a representative 6 hour live video microscopy of live *C. albicans* being ingested by macrophages. It further illustrates hyphal growth within macrophages and macrophage killing by hyphal *C. albicans*.
(MP4)

Video S2 **Phagocytosis of live C. albicans by J774.1 macrophages.** Shows a high magnification (×80) representative 6 hour live video microscopy of live *C. albicans* being ingested by macrophages. It further illustrates hyphal growth within macrophages and macrophage killing by hyphal *C. albicans*.
(MP4)

Video S3 **Macrophage tracking of C. albicans.** Shows an example of a J774.1 macrophage tracking a UV-killed C.albicans yeast cell. It illustrates random macrophage movement followed by directional tracking of the fungal cell and ultimately uptake of the pathogen.
(MP4)

Video S4 **Projection movie of** ***C. albicans*** **phagocytosis by macrophages.** Shows a 3D projection of *C. albicans* ingested by macrophages. It also confirms co-localisation of green FITC stained fungal cells with lysotracker red in macrophage phagosomes.
(MOV)

## References

[1] Bistoni F, Vecchiarelli A, Cenci E, Puccetti P, Marconi P (1986). Evidence for macrophage-mediated protection against lethal *Candida albicans* infection.. Infect Immun.

[2] Netea MG, Brown GD, Kullberg BJ, Gow NA (2008). An integrated model of the recognition of *Candida albicans* by the innate immune system.. Nat Rev Microbiol.

[3] Sheth CC, Hall R, Lewis L, Brown AJ, Odds FC (2011). Glycosylation status of the *C. albicans* cell wall affects the efficiency of neutrophil phagocytosis and killing but not cytokine signaling.. Med Mycol.

[4] Kapteyn JC, Hoyer LL, Hecht JE, Muller WH, Andel A (2000). The cell wall architecture of *Candida albicans* wild-type cells and cell wall-defective mutants.. Mol Microbiol.

[5] Cutler JE (2001). N-glycosylation of yeast, with emphasis on *Candida albicans*.. Med Mycol.

[6] Ernst JF, Prill SK (2001). O-glycosylation.. Med Mycol.

[7] Moyes DL, Runglall M, Murciano C, Shen C, Nayar D (2010). A biphasic innate immune MAPK response discriminates between the yeast and hyphal forms of *Candida albicans* in epithelial cells.. Cell Host Microbe.

[8] Cheng SC, van de Veerdonk FL, Lenardon M, Stoffels M, Plantinga T (2011). The dectin-1/inflammasome pathway is responsible for the induction of protective T-helper 17 responses that discriminate between yeasts and hyphae of *Candida albicans*.. J Leukoc Biol.

[9] Kumamoto CA, Vinces MD (2005). Contributions of hyphae and hypha-co-regulated genes to *Candida albicans* virulence.. Cell Microbiol.

[10] McKenzie CG, Koser U, Lewis LE, Bain JM, Mora-Montes HM (2010). Contribution of *Candida albicans* cell wall components to recognition by and escape from murine macrophages.. Infect Immun.

[11] Brown GD (2011). Innate antifungal immunity: The key role of phagocytes.. Annu Rev Immunol.

[12] Kaposzta R, Marodi L, Hollinshead M, Gordon S, da Silva RP (1999). Rapid recruitment of late endosomes and lysosomes in mouse macrophages ingesting *Candida albicans*.. J Cell Sci.

[13] Netea MG, Marodi L (2010). Innate immune mechanisms for recognition and uptake of *Candida* species.. Trends Immunol.

[14] Netea MG, Gow NA, Munro CA, Bates S, Collins C (2006). Immune sensing of *Candida albicans* requires cooperative recognition of mannans and glucans by lectin and toll-like receptors.. J Clin Invest.

[15] Means TK, Mylonakis E, Tampakakis E, Colvin RA, Seung E (2009). Evolutionarily conserved recognition and innate immunity to fungal pathogens by the scavenger receptors SCARF1 and CD36.. J Exp Med.

[16] Miyazato A, Nakamura K, Yamamoto N, Mora-Montes HM, Tanaka M (2009). Toll-like receptor 9-dependent activation of myeloid dendritic cells by deoxynucleic acids from *Candida albicans*.. Infect Immun.

[17] McGreal EP, Rosas M, Brown GD, Zamze S, Wong SY (2006). The carbohydrate-recognition domain of dectin-2 is a C-type lectin with specificity for high mannose.. Glycobiology.

[18] Wells CA, Salvage-Jones JA, Li X, Hitchens K, Butcher S (2008). The macrophage-inducible C-type lectin, mincle, is an essential component of the innate immune response to *Candida albicans*.. J Immunol.

[19] Mora-Montes HM, Netea MG, Ferwerda G, Lenardon MD, Brown GD (2011). Recognition and blocking of innate immunity cells by *Candida albicans* chitin.. Infect Immun.

[20] Keppler-Ross S, Douglas L, Konopka JB, Dean N (2010). Recognition of yeast by murine macrophages requires mannan but not glucan.. Eukaryot Cell.

[21] Munro CA, Bates S, Buurman ET, Hughes HB, Maccallum DM (2005). Mnt1p and Mnt2p of *Candida albicans* are partially redundant alpha-1,2-mannosyltransferases that participate in O-linked mannosylation and are required for adhesion and virulence.. J Biol Chem.

[22] Mora-Montes HM, Bates S, Netea MG, Diaz-Jimenez DF, Lopez-Romero E (2007). Endoplasmic reticulum alpha-glycosidases of *Candida albicans* are required for N glycosylation, cell wall integrity, and normal host-fungus interaction.. Eukaryot Cell.

[23] Hobson RP, Munro CA, Bates S, MacCallum DM, Cutler JE (2004). Loss of cell wall mannosylphosphate in *Candida albicans* does not influence macrophage recognition.. J Biol Chem.

[24] Zheng X, Wang Y, Wang Y (2004). Hgc1, a novel hypha-specific G1 cyclin-related protein regulates *Candida albicans* hyphal morphogenesis.. EMBO J.

[25] Lo HJ, Kohler JR, DiDomenico B, Loebenberg D, Cacciapuoti A (1997). Nonfilamentous *C. albicans* mutants are avirulent.. Cell.

[26] McPhillips KA, Erwig LP (2009). Assessment of apoptotic cell phagocytosis by macrophages.. Methods Mol Biol.

[27] Hobson RP, Munro CA, Bates S, MacCallum DM, Cutler JE (2004). Loss of cell wall mannosylphosphate in *Candida albicans* does not influence macrophage recognition.. J Biol Chem.

[28] Klippel N, Cui S, Groebe L, Bilitewski U (2010). Deletion of the *Candida albicans* histidine kinase gene CHK1 improves recognition by phagocytes through an increased exposure of cell wall beta-1,3-glucans.. Microbiology.

[29] Mora-Montes HM, Bates S, Netea MG, Castillo L, Brand A (2010). A multifunctional mannosyltransferase family in *Candida albicans* determines cell wall mannan structure and host-fungus interactions.. J Biol Chem.

[30] Erwig LP, McPhilips KA, Wynes MW, Ivetic A, Ridley AJ (2006). Differential regulation of phagosome maturation in macrophages and dendritic cells mediated by rho GTPases and ezrin-radixin-moesin (ERM) proteins.. Proc Natl Acad Sci U S A.

[31] McKenzie CG, Koser U, Lewis LE, Bain JM, Mora-Montes HM (2010). Contribution of *Candida albicans* cell wall components to recognition by and escape from murine macrophages.. Infect Immun.

[32] Jouault T, Fradin C, Trinel PA, Bernigaud A, Poulain D (1998). Early signal transduction induced by *Candida albicans* in macrophages through shedding of a glycolipid.. J Infect Dis.

[33] Mora-Montes HM, Bates S, Netea MG, Castillo L, Brand A (2010). A multifunctional mannosyltransferase family in *Candida albicans* determines cell wall mannan structure and host-fungus interactions.. J Biol Chem.

[34] Buurman ET, Westwater C, Hube B, Brown AJ, Odds FC (1998). Molecular analysis of CaMnt1p, a mannosyl transferase important for adhesion and virulence of *Candida albicans*.. Proc Natl Acad Sci U S A.

[35] Bates S, MacCallum DM, Bertram G, Munro CA, Hughes HB (2005). *Candida albicans* Pmr1p, a secretory pathway P-type Ca2+/Mn2+-ATPase, is required for glycosylation and virulence.. J Biol Chem.

[36] Bates S, Hughes HB, Munro CA, Thomas WP, MacCallum DM (2006). Outer chain N-glycans are required for cell wall integrity and virulence of *Candida albicans*.. J Biol Chem.

[37] Lu Y, Su C, Wang A, Liu H (2011). Hyphal development in *Candida albicans* requires two temporally linked changes in promoter chromatin for initiation and maintenance.. PLoS Biol.

[38] Gow NA, Brown AJ, Odds FC (2002). Fungal morphogenesis and host invasion.. Curr Opin Microbiol.

[39] Sudbery PE (2011). Growth of *Candida albicans* hyphae.. Nat Rev Microbiol.

[40] Kadosh D, Johnson AD (2005). Induction of the *Candida albicans* filamentous growth program by relief of transcriptional repression: A genome-wide analysis.. Mol Biol Cell.

[41] Saville SP, Lazzell AL, Monteagudo C, Lopez-Ribot JL (2003). Engineered control of cell morphology in vivo reveals distinct roles for yeast and filamentous forms of *Candida albicans* during infection.. Eukaryot Cell.

[42] Marcil A, Gadoury C, Ash J, Zhang J, Nantel A (2008). Analysis of PRA1 and its relationship to *Candida albicans*- macrophage interactions.. Infect Immun.

[43] d'Ostiani CF, Del Sero G, Bacci A, Montagnoli C, Spreca A (2000). Dendritic cells discriminate between yeasts and hyphae of the fungus *Candida albicans*. Implications for initiation of T helper cell immunity in vitro and in vivo.. J Exp Med.

[44] Monks J, Henson PM (2009). Differentiation of the mammary epithelial cell during involution: Implications for breast cancer.. J Mammary Gland Biol Neoplasia.

[45] Champion JA, Mitragotri S (2006). Role of target geometry in phagocytosis.. Proc Natl Acad Sci U S A.

[46] Murad AM, Lee PR, Broadbent ID, Barelle CJ, Brown AJ (2000). CIp10, an efficient and convenient integrating vector for *Candida albicans*.. Yeast.

